# Parental post-traumatic stress and psychiatric care utilisation among refugee adolescents

**DOI:** 10.1007/s00787-021-01827-1

**Published:** 2021-06-19

**Authors:** Lisa Berg, Edith de Montgomery, Monica Brendler-Lindquist, Ellenor Mittendorfer-Rutz, Anders Hjern

**Affiliations:** 1 Department of Public Health Sciences, Stockholm University, Stockholm, Sweden; 2 Centre for Health Equity Studies, Stockholm University/Karolinska Institutet, Stockholm, Sweden; 3 Department of Public Health, Danish Institute for Migration, Ethnicity and Health, University of Copenhagen, Copenhagen, Denmark; 4 Former Director, the Red Cross Centre for Tortured Refugees, Stockholm, Sweden; 5 Department of Insurance Medicine, Karolinska Institutet, Stockholm, Sweden; 6 Clinical Epidemiology, Department of Medicine, Karolinska Institutet, Stockholm, Sweden

**Keywords:** Refugees, Parental post-traumatic stress, Intergenerational trauma, Psychiatric care, Adolescents

## Abstract

Parental psychiatric morbidity related to experiences of war and trauma has been associated with adverse psychological outcomes for children. The aim of this study was to investigate parental post-traumatic stress in relation to psychiatric care utilization among children of refugees with particular attention on the child’s own refugee status, sex of both child and parents, and specific psychiatric diagnoses. This was a register study in a population of 16 143 adolescents from refugee families in Stockholm County born 1995–2000 and followed between 2011 and 2017 (11–18 years old). Parental post-traumatic stress, identified in three levels of care, was analysed in relation to child and adolescent psychiatric care use. Cox regression analysis was used to estimate hazard ratios (HR) and 95% confidence intervals (CI), adjusted for duration of residence and demographic and socioeconomic variables. Having a mother with post-traumatic stress was associated with higher psychiatric care utilization, with adjusted HR 2.44 (95% CI 1.90–3.14) among foreign-born refugee children and HR 1.77 (1.33–2.36) among Swedish-born children with refugee parents, with particularly high risks for children with less than five years of residence (HR 4.03; 2.29–7.10) and for diagnoses of anxiety and depression (HR 2.71; 2.11–3.48). Having a father with post-traumatic stress was not associated with increased HRs of psychiatric care utilization. Similar results were seen for boys and girls. Treatment for post-traumatic stress should be made available in refugee reception programmes. These programmes should use a family approach that targets both parents and children.

**Supplementary Information:**

The online version contains supplementary material available at 10.1007/s00787-021-01827-1.

## Introduction

An increasing number of people around the world are being forced to flee their home countries due to armed conflicts, organized violence, and persecution. During the past decades, the number of refugees seeking asylum in Sweden and other European countries has increased substantially [1]. Traumatic experiences of violence and torture combined with adverse social and economic circumstances in the new country contribute to an increased susceptibility to mental ill health, and refugees in exile have been shown to have high prevalence rates of psychiatric disorders, in particular post-traumatic stress disorders [2, 3].

High levels of mental ill health have also been seen in refugee children, particularly during the first years after settlement [4, 5]. However, previous studies have demonstrated a lower use of child and adolescent psychiatric services among refugees, particularly among newly arrived refugee children and children with a background in low- and middle-income countries [6, 7]. Although use of psychiatric services increases the longer children are in their new country, indicating that many barriers to care decrease with time, previous studies have highlighted several cultural and structural barriers to accessing treatment [8].

As for adult refugees, the increase in mental health problems among foreign-born refugee children is most likely associated with difficult experiences in the country of origin and during migration combined with the conditions in the new country. Previous studies have highlighted the importance of family-related problems and parental difficulties related to experiences of war and trauma for psychological outcomes among children in refugee families [9–11]. However, recent studies have been inconclusive with respect to psychiatric hospital contacts in children of refugee parents with psychological difficulties after traumatic experiences [12, 13]. One study demonstrated an increased risk of psychiatric hospital contacts in refugee children who had a parent diagnosed with post-traumatic stress disorder (PTSD) [12], but another recent study using information from treatment centres specializing in traumatized refugees found no increased risk of psychiatric morbidity in children who had a parent exposed to trauma [13]. Thus, previous studies are few, findings are contradictory, and few studies use complementary sources of information such as hospitalization data combined with primary care and specialized treatment centre data. Furthermore, there is little evidence on specific types of mental health problems and whether there are differences in diagnostic patterns (e.g., in relation to exposure of parental post-traumatic stress and between native- and foreign-born children). Increased understanding of these associations and patterns will help identify individuals in need of treatment and how to prioritize resources to prevent intergenerational consequences of psychological trauma.

Importantly, since refugee children with parents who suffer from post-traumatic stress probably have a high risk themselves of having been exposed to traumatic experiences that could lead to an increased need for psychiatric treatment, children born in exile need to be analysed separately from children who are themselves refugees. Furthermore, in a recent study, we demonstrate that psychiatric care use is particularly low in newly arrived refugee adolescents, particularly when the families are from low- and middle-income countries [7], which emphasizes the importance of considering duration of residence and the developmental level of the family’s country of origin.

Against this background, we investigated utilization of child and adolescent psychiatric care services among children of refugee parents with post-traumatic stress using combined information from specialized treatment centres for refugees as well as primary and specialist care. In these analyses, we considered socioeconomic factors and important determinants of care utilization among refugee children, including the developmental level of parental country of origin and duration of residence. We give particular attention to the importance of the child’s own refugee status, the sex of both child and parents, as well as specific psychiatric diagnoses.

## Methods

### Data material and study population

Data from Swedish national registers were combined with regional data on healthcare use. The Swedish Register of the Total Population [14] was used to identify the study population, which consisted of 16 143 boys and girls (in 11,813 families) born between 1995 and 2000 and who resided in Stockholm County the year of their 15th birthday. The children were linked to their parents through the Multi-Generation Register [15] using the personal identity number assigned to all Swedish residents at birth or upon immigration. Before data are made available for research purposes, the personal identification numbers are replaced with random reference numbers, so all data are analysed anonymously.

### Refugee background

Data on refugee status, including information on year of immigration and reason for obtaining residency for the children and their parents, were retrieved from the Longitudinal Database for Integration Studies (STATIV, Swedish acronym) developed by Statistics Sweden. The information was used to identify children with a refugee background (i.e., children who themselves had been granted residence permit as a refugee or who had at least one refugee parent). Foreign-born refugee children were further categorized as family reunification or asylum refugees (including quota refugees), which was determined by identifying the reason for obtaining residency.

### Parental post-traumatic stress

Information on parental post-traumatic stress was captured by a diagnosis of PTSD (International Classification of Diseases (ICD)-10 code F43.1) or a diagnosis of Enduring Personality Change after Catastrophic Experience (ICD-10 code F62.0) from three levels of care: (1) primary care (either of these two diagnoses recorded by a primary care physician in the regional health care database in the Stockholm region, 2006–2017); (2) secondary care (either of these two diagnoses from a psychiatric clinic recorded in the regional health care database in the Stockholm region, 2006–2017); and (3) a previous patient at The Red Cross Centre for Tortured Refugees in Stockholm, 2006–2014 (information retrieved from The Red Cross Centre for Tortured Refugees). All primary and secondary care visits financed by the Stockholm County Council are included in the health care database, with the exception of visit with a private specialist not funded by the region. In a recent validation of PTSD diagnoses in the regional health care database in the Stockholm region against patient records, quality for epidemiological research was found to be acceptable [16]. The diagnosis of Enduring Personality Change after Catastrophic Experience has recently been created to capture the more chronic personality changes seen after severe and repeated trauma, such as torture [17], and was for this reason included in the definition of post-traumatic stress*.*

### Child and adolescent psychiatric care

Use of child and adolescent psychiatric services was defined as at least one visit registered in the regional health care database in the Stockholm region between 2011 and 2017. This database includes all visits to child and adolescent psychiatric services in the region irrespective of whether the healthcare provider was public or private. The upper age limit for child and adolescent psychiatry in Stockholm is 18 years, and the study population was followed between 3 and 7 years between the ages of 11 and 18 years. The health care database includes all child psychiatric services in the Stockholm region. The main type of mental health problem was categorized based on the main diagnosis at last visit during the follow-up period per the WHO ICD-10 classification [18]. The diagnoses were categorized into three overarching groups: (1) Neuropsychiatric conditions, with ADHD (F90), autism-spectrum disorders (F84), intellectual disabilities (F70–F79, F849), and obsessive compulsive disorder (F42) as the major diagnostic groups; (2) Diagnoses in the anxiety and depression chapters (F32–F41, F43–F48), with anxiety syndrome (F41), depression (F32–F34, F39), severe stress (F430, F432, F438, F439), PTSD (F431), and phobic syndromes (F40) as the diagnostic items; and (3) Other, with eating disorders (F50), conduct disorders/ODD (F91), bipolar (F30, F31), schizophrenia (F2), and substance use disorders (F1) as the diagnostic items.

### Covariates

Demographic data on date of birth and sex were retrieved from the Register of the total Population. Information on parents’ highest achieved educational level, categorized as compulsory school or less (0–9 years), upper secondary school (10–13 years), or university (13 + years), was retrieved from the Swedish national longitudinal integrated database for health insurance and labor market studies for 2011 [19]. The Register of the Total Population provided information on country or region of birth of both children and parents. Country of origin (i.e., the mother’s country of birth) was categorized as low, middle, or high-income countries according to the World Bank classification from 2011 [20]. When this information was missing, the father’s country of origin was used. Duration of residence in Sweden was defined as years since receiving residence permit at start of follow-up in 2011 for foreign-born parents and at age 16 for foreign-born children (the age when duration of residence could be measured at the same age for all birth cohorts). Family status was coded as single parent household (yes/no).

### Statistical analysis

Cox proportional hazard models were used to estimate hazard ratios (HR) and 95% confidence intervals (CI) for psychiatric health care utilization in relation to parental post-traumatic stress. Person-time of follow-up was accumulated from 1 January 2011 to the date of the first visit to a psychiatric clinic, the date of their 18th birthday, or 31 December 2017, whichever came first. All analyses were stratified by sex of the child and interaction analyses were performed to investigate statistically significant differences. If there were no differences, the results were presented for boys and girls combined. Chi-squared tests were performed to assess differences in main diagnoses between children with and without experience of parental post-traumatic stress. *P* values are presented in Table 5.

In the regression analyses, Model 1 was adjusted for birth year and sex, and Model 2 was adjusted additionally for potentially confounding factors (i.e., parental educational level, duration of residence, and parental country of origin). Model 3 added single parenthood, which may be considered as a potential mediator of the association. Robust standard errors were used to account for sibling correlation. A sensitivity analysis restricting the population to children who had two recorded parents in the Multi-Generation register was carried out to investigate the potential influence of missing parents in analyses adjusting for single parent households. SAS version 9.4 (SAS Institute Inc., Cary, NC, USA) was used for all analyses.

## Results

Of the 16 143 adolescents, 47% were foreign-born and had been granted residence permits as refugees, and 53% were Swedish-born with refugee parents. The majority of the parents (80%) had a university or upper secondary level education, and there were only minor differences between families with and without experience of parental post-traumatic stress (Table 1). More than half (59%) of the refugee parents had been living in Sweden for more than 10 years at start of follow-up. The vast majority of the parents were from low (27%) or middle (69%) income countries, mainly the Horn of Africa, Iraq, Iran, and Syria (Table S1).

**Table 1:** Characteristics of the study population born between 1995 and 2000

	All	No parental post-traumatic stress	Parental post-traumatic stress
	%	%	%
No. (% of total)	16143	14 330 (88.8%)	1 813 (11.2%)
Sex			
Girls	49.2	49.4	47.2
Boys	50.8	50.6	52.8
Duration of residence at age 16			
Born in Sweden	52.7	53.3	47.9
10 + years	14.5	14.0	18.3
6–9 years	23.8	23.4	27.4
0–5 years	9.0	9.3	6.4
Parents highest education			
University	43.9	43.8	44.4
Upper secondary school	35.9	36.3	32.3
Compulsory school or less	20.3	19.9	23.4
Parental country of origin			
High income	4.1	4.5	1.0
Middle income	69.3	67.6	82.0
Low income	26.6	27.8	17.0
Single parent household			
No	68.3	68.5	66.7
Yes	31.7	31.5	33.3

Numbers are column percentages unless otherwise stated

In total, 10.2% of the Swedish-born adolescents and 12.4% of the foreign-born adolescents had at least one parent treated for post-traumatic stress. Proportions of children with parents treated for post-traumatic stress were slightly higher for fathers (5.3%) compared with mothers (4.7%) treated for post-traumatic stress, and for children with parents from middle- (6.6%) and low-income countries (2.4%) compared with high-income countries (0.6%) (Table 2).

**Table 2:** Proportions of children with parents treated for post-traumatic stress by covariates

	Father post-traumatic stress	Mother post-traumatic stress	Both parents post-traumatic stress
	%	%	%
All children (no.)	5.3 (851)	4.7 (759)	1.3 (203)
Parents highest education			
University	5.1	4.8	1.5
Upper secondary school	5.0	4.1	1.0
Compulsory school or less	6.1	5.6	1.3
Parental country of origin			
High income country	0.6	1.8	0.3
Middle income country	6.6	5.2	1.5
Low income country	2.4	4.0	0.7
Single parent household			
No	5.7	3.9	1.4
Yes	4.7	6.5	0.9
Duration of residence at age 16			
Born in Sweden	5.1	4.3	0.8
10 + years	7.3	4.8	2.1
6–9 years	5.4	5.7	1.9
0–5 years	2.9	4.2	1.0

Use of psychiatric care services was slightly more common among the Swedish-born (9.6%) than non-Swedish-born adolescents (8.4%). Post-traumatic stress in mothers, but not fathers, was clearly associated with more use of psychiatric care (Table 3). Overall, psychiatric care was used less by adolescents from families originating in low- or middle-income countries, adolescents with a shorter duration of residence, and adolescents who settled in Sweden in family reunification with refugee parents. However, psychiatric services were more common for adolescents in single parent households. Small differences were seen in type of care for mother and fathers with post-traumatic stress, with the highest levels for specialist psychiatric care (Table 3).

**Table 3:** Percentage with at least one visit to child and adolescent psychiatric services by exposure to parental post-traumatic stress and covariates

	All	No post-traumatic stress	Father post-traumatic stress	Mother post-traumatic stress
	*n* = 16 143	*n* = 14 330	*n* = 851	*n* = 759
	%	%	%	%
All	9.0	8.5	8.7	17.5
Sex				
Girls	9.4	8.9	9.9	17.8
Boys	8.6	8.2	7.7	17.3
Parental educational level				
Compulsory school or less	7.6	7.0	6.5	15.8
Upper secondary school	9.2	9.0	8.6	15.3
University	9.5	8.8	9.9	20.1
Parental country of origin				
High income	14.4	14.0	25.0	33.3
Middle income	9.5	9.1	8.8	17.2
Low income	6.9	6.4	7.6	17.5
Duration of residence at age 16				
Born in Sweden	9.6	9.2	10.4	16.8
10 + years	9.8	9.2	7.6	20.5
6–9 years	8.1	7.6	6.8	16.1
0–5 years	6.9	6.2	4.8	21.7
Ground for residency of child				
Swedish-born	9.6	9.2	10.4	16.8
Asylum and quota refugees	10.1	9.4	10.7	21.3
Family reunification	5.1	4.6	4.4	12.1
Single parent household				
No	7.3	7.0	6.9	12.1
Yes	12.8	11.8	13.6	24.6
Type of care				
Red cross centre	–	–	8.5	16.1
Specialist psychiatric care	–	–	10.6	18.2
Primary care	–	–	7.8	13.1

Post-traumatic stress in mothers was associated with increased HRs of psychiatric care, whereas no association was seen for post-traumatic stress in fathers (Table 4). HRs in relation to post-traumatic stress in mothers remained elevated in models adjusted for parental educational levels and migration-related factors (HR 2.25, 95% CI 1.86–2.73). Estimates were slightly attenuated in models adjusted for a potentially mediating effect of single parenthood (HR 2.09, 95% CI 1.72–2.52). In analyses restricted to adolescents in non-separated families, HRs of psychiatric care utilization in relation to paternal post-traumatic stress remained non-elevated (HR 1.01, 95% CI 0.74–1.39).

**Table 4:** Cox regression of any use of psychiatric health care services during the study period

	Model 1^a^	Model 2^b^	Model 3^c^
	HR (95% CI)	HR (95% CI)	HR (95% CI)
Parental post-traumatic stress			
No post-traumatic stress	1	1	1
Father post-traumatic stress	1.03 (0.81–1.31)	1.01 (0.79–1.29)	1.01 (0.79–1.29)
Mother post-traumatic stress	2.22 (1.84–2.69)	2.25 (1.86–2.73)	2.09 (1.72–2.52)
Both parents	1.37 (0.92–2.05)	1.37 (0.92–2.06)	1.44 (0.96–2.14)

^a^Model 1 adjusted for birth year and sex

^b^Model 2 adjusted additionally for parental educational level, duration of residence, and parental country of origin

^c^Model 3 adjusted additionally for single parent household

HRs in relation to having a mother with post-traumatic stress were similarly elevated among girls (HR 2.17, 95% CI 1.67–2.83) and boys (HR 2.12, 95% CI 1.61–2.78), and there were no statistically significant interaction (*P* = 0.846). Elevated estimates were seen among the foreign-born refugee adolescents (HR 2.44, 95% CI 1.90–3.14) as well as among Swedish-born (HR 1.77, 95% CI 1.33–2.36). HRs from sensitivity analyses where the population was restricted to the 82.1% who had two parents recorded in the Swedish national registers were very similar to the estimates from the main analyses (Table S3).

Interaction analyses revealed a statistically significant interaction between duration of residence (0–5 years versus 6 + years) and post-traumatic stress in mothers (*P* = 0.04). Among foreign-born adolescents with a shorter duration of residence (≤ 5 years at age 16), post-traumatic stress in a mother was associated with particularly high HR for psychiatric care (HR 4.03, 95% CI 2.29–7.10). HR for psychiatric care among those with longer duration was 2.16 (95% CI 1.68–2.78).

About half (52%) of the mothers with post-traumatic stress had been treated in specialist psychiatric care, 32% in primary care, and 15% at The Red Cross Centre for Tortured Refugees. Regression analyses revealed minor differences between type/level of care. Children with a mother treated for post-traumatic stress in specialist psychiatric care or in treatment centre for torture victims were more than twice as likely to have used child and adolescent psychiatric services, whereas HR for psychiatric care for children with a mother treated for post-traumatic stress in primary care was somewhat lower (Table S3).

An overview of main diagnoses at last visit during the study period is presented in Table 5. Clear differences were seen between Swedish-born children with refugee parents and foreign-born refugee children, with a particularly high proportion of diagnoses in the depression and anxiety chapters among the foreign-born individuals. This group also exhibited clear differences between adolescents with and without a parent who had experienced post-traumatic stress. In the group of foreign-born refugee children, adjusted HR was 2.71 for having a diagnosis in the anxiety and depression chapters in children of mothers with post-traumatic stress (95% CI 2.11–3.48; not in table).

**Table 5:** Main diagnosis in psychiatric patients at last visit to the clinic during the study period by birthplace and exposure to parental post-traumatic stress (*N* = 1 456)

	Swedish-born		Foreign-born	
	No parental post-traumatic stress	Parental post-traumatic stress	No parental post-traumatic stress	Parental post-traumatic stress
	*N* = 702	*N* = 114	*N* = 524	*N* = 116
	%	%	%	%
Neuropsychiatric conditions				
ADHD	18.1	24.6	16.4	10.3
Autism-spectrum disorders	9.8	5.3	4.0	1.7
Intellectual disabilities	6.3	7.0	3.8	3.5
Obsessive–compulsive disorder (OCD) (F42)	2.4	1.8	1.5	0.9
All	*36.6*	*38.6* (*p* = *0.683*)	*25.8*	*16.4* (*p* = *0.032*)
Diagnoses in the anxiety and depression chapters				
Anxiety syndrome	18.2	13.2	15.5	16.4
Depression	13.3	13.2	12.4	18.1
Severe stress, excluding PTSD	4.3	6.1	13.4	19.0
PTSD	2.1	2.6	5.3	10.3
Phobic syndromes	2.0	1.8	1.9	1.7
Other	3.0	1.8	3.2	0.9
All	*42.9*	*38.6* (*p* = *0.391*)	*51.7*	*66.4* (*p* = *0.004*)
Other				
Eating disorders	2.1	0.9	1.7	0.9
Conduct/ODD	3.4	7.0	4.0	2.6
Bipolar, schizophrenia, substance us14 e	2.3	0.9	1.9	–
Uncategorised	12.7	14.0	14.9	13.8
All	*20.5*	*22.8* (*p* = *0.576*)	*22.5*	*17.2* (*p* = *0.211*)

Column percentages. *P* values from chi-squared tests

## Discussion

The findings from this study demonstrate that post-traumatic stress in mothers, but not fathers, was associated with increased use of psychiatric services by foreign-born refugee children as well as in Swedish-born children with refugee parents. Particularly high risks were seen in more recently arrived refugee adolescents and for diagnoses of introverted problems.

In the few previous studies on consequences of parental post-traumatic stress for psychiatric care contacts among refugee children, the findings have been conflicting. A recent Danish study reported no risk differences between children of parents who had been treated for war or torture trauma at specialist centres and non-exposed children of parents originating from the same region [13]. This apparent lack of association between parental trauma exposure and offspring psychiatric disorders may be explained by misclassification of exposure, diluting the association, a limitation also mentioned by the authors. Other potential explanations of these findings include important post-migration factors. Consistent with our findings [7] and previous findings [6, 21], children of foreign-born parents in this Danish study had fewer hospital contacts, indicating an underutilisation of mental health services, the existence of barriers to care, and an unmet need for healthcare, rather than lower levels of mental health problems. Another recent Danish study demonstrated an increased risk of psychiatric hospital contacts related to parental diagnosis of PTSD, defined by hospital contacts only [12]. This study demonstrated an increased risk associated with a diagnosis of PTSD in fathers as well as mothers, contrasting with our findings with regards to fathers. Our study, which includes information on maternal and paternal post-traumatic stress diagnosed on three levels of care and information from child and adolescent psychiatric services, provides further support for the significance of intergenerational effects of psychological trauma among refugees in exile.

The majority of previous studies on correlations between parental and children’s psychopathology has focused solely on mother–child dyads [22]. One previous study investigating quantity and quality of father involvement and the influence of post-traumatic stress in a population of refugees and asylum seekers in the Netherlands found fathers to be less involved than mothers in caregiving interactions, but no differences were seen in quality of parent–child involvement [23]. Even considering fathers increased involvement as caregivers, if the mother is still more often the primary caregiver this could potentially contribute to the explanation of the gender difference observed in our study. Furthermore, the authors of the Dutch study argue that fathers might have more opportunities to withdraw from family-interaction when symptoms of stress worsen and by doing so diminishing the negative impact of trauma-related stress on their children [23]. It can also be hypothesized, as a contributing explanation to this difference, that traumatic experiences that are shared by parent and child are more common for mothers and children than for fathers and children. Given the conflicting findings in existing studies and the important questions these findings raise, further studies are needed to clarify these patterns, preferably studies that include interviews with parents and children.

The diagnostic patterns described in Table 5 show a similar pattern with and without exposure to parental post-traumatic stress in the Swedish-born patients. In contrast, a very clear diagnostic pattern was found in the foreign-born patients exposed to maternal post-traumatic stress with a twofold elevated risk of having a diagnosis in the anxiety and depression chapters. A similar high burden of introverted psychiatric symptoms has been found in multiple Scandinavian studies of refugee children and adolescents during the first years after settlement [9]. These symptoms have been described to be associated with the children’s own exposure to organized violence and migration stress, with the exposure often being shared by children and their mothers. Thus, we could not detect any specific diagnostic pattern associated with exposure to parental post-traumatic stress in this study.

A particularly high use of psychiatric services in relation to maternal post-traumatic stress was seen among adolescents with a shorter duration of residence. Although previous studies have demonstrated an underutilisation of mental health services, particularly during the first years after settlement, longitudinal studies in Scandinavia have also shown very high prevalence of poor mental health shortly after settlement that tended to decrease with increasing duration of residence [9, 24, 25].

The increased use of psychiatric services among children of parents treated for post-traumatic stress was not limited to foreign-born children. Thus, an increased need for post-traumatic psychiatric treatment following their own experiences among children who were themselves refugees did not fully explain these associations. Although few studies have examined children of refugees, intergenerational transmission of trauma has been investigated at length in clinical studies with offspring of Holocaust survivors and among children of soldiers [26, 27]. Previous findings have suggested that psychological and social consequences of traumatic experiences may affect health and well-being of the children [9, 10]. Healthy development and psychological well-being of a child are closely linked to the health of the caregiver, and a positive relationship between child and caregiver is crucial. The literature suggests different mechanisms in the intergenerational transmission of trauma, mainly focusing on parental trauma-related mental health problems, caregiving behavior, and disrupted attachment [28, 29]. Caregiver mental health problems following traumatic experiences may have adverse effects on parents’ emotional and behavioral availability, undermine the parents’ ability to provide support and a sense of security for their children and have consequences for parent–child interaction and attachment [30, 31]. Well-being and mental health of their children may also be negatively affected as a result of poor family functioning and instability and parenting style [11, 31, 32]. Previous studies have also highlighted increased risks of harsh parenting and family violence in families affected by post-traumatic stress [33, 34]. Future research should focus on these mechanisms or pathways through which these associations are mediated.

### Strengths and limitations

A major strength of the present study is the availability of data from specialized treatment centres and primary care, in addition to the previously validated data on post-traumatic stress from specialist care as the use of hospitalization data only might underestimate psychiatric contacts. Other strengths include the use of register data and the large study population comprising all refugee children and adolescents residing in the county of Stockholm between 2011 and 2017. The data from reliable national and local registers allowed for adjustment for a wide range of potentially confounding or mediating variables, including socioeconomic factors and migration-related variables.

The prevalence of post-traumatic stress in refugee parents in this study was lower compared to what has been seen in many adult refugee populations [2]. Previous research has highlighted several barriers to psychiatric care [8, 35], suggesting that not everyone suffering from psychological consequences of trauma seeks medical attention, which could potentially lead to an underestimation of the affected parents.

Child psychiatric services in Stockholm, the largest of Sweden’s health care organizations, has considerable resources. These services are free and can be arranged through self-referral from parents as well as schools. Child psychiatric services are probably more accessible than in many other contexts, and as many as 10% of adolescents in Stockholm between 13 and 17 years old have at least one contact with these services annually [36].

One limitation of this study is that the register data do not include information on children in asylum-seeking families and children of undocumented parents. Other limitations are the lack of information on actual health status and health care need in the registries and the potential risk of detection bias (i.e., children with parents receiving care may be more likely to receive care themselves, resulting in a potential overestimation of the associations).

### Implications

The findings from this study, based on data for more than 16000 children with a refugee background, demonstrated that refugee adolescents with a mother treated for post-traumatic stress were more than twice as likely to have been in contact with child and adolescent psychiatric services. These findings emphasize that efforts to improve mental health of refugee children and adolescents need to include mental health interventions across generations, ensuring that parents in need of post-traumatic psychiatric treatment and their children are offered correct treatment and support. As demonstrated previously [7, 21], refugee children and adolescents are less likely to use psychiatric healthcare services compared with their majority population peers. For refugee adolescents to access appropriate health care services when such services are needed, there is a need to address the different types of barriers to care. It is also important to acknowledge the important role of schools [37] in identifying mental health issues in refugee children and adolescences and facilitating access to mental health services.

## Supplementary Information

Below is the link to the electronic supplementary material.

Supplementary file1 (DOCX 24 KB)

## Data Availability

The data used in this article cannot be shared publicly since sharing of data is restricted by Swedish data protection laws and administrative data are made available for specific research projects. Thus, the data used for this study cannot be shared with other researchers.
