# Supplementary material for: Parental post-traumatic stress and psychiatric care utilisation among refugee adolescents
Source: Eur Child Adolesc Psychiatry. 2021 Jun 19;31(12):1953–62. doi: 10.1007/s00787-021-01827-1 (PMC9663346; doi:10.1007/s00787-021-01827-1)
Supplement: Supplementary file 1 — Supplementary file1 (DOCX 24 KB) [file 787_2021_1827_MOESM1_ESM.docx]

## Table S1. Parental origin by country income level of the refugee families, % (no.)

| **High income** |  |
| --- | --- |
| 1. Europe | 2.1 (347) |
| 2. East Asia | 1.0 (160) |
| 3. North America | 1.0 (159) |
| *All* | *4.1 (666)* |
| **Middle income countries** |  |
| 1. Iraq | 24.5 (3 959) |
| 2. Syria | 7.1 (1 152) |
| 3. Asia (not East or South Asia) | 6.7 (1 076) |
| 4. Iran | 6.6 (1 067) |
| 5. South America | 6.1 (988) |
| 6. Africa (not Somalia, Eritrea or Ethiopia) | 5.6 (898) |
| 7. Former Yugoslavia | 4.8 (768) |
| 8. Lebanon | 2.0 (317) |
| 8. Other (other European, | 5.9 (954) |
| *All* | *69.3 (11 179)* |
| **Low income countries** |  |
| 1. Somalia | 11.6 (1 879) |
| 2. Eritrea or Ethiopia | 8.2 (1 328) |
| 3. Afghanistan | 3.8 (607) |
| 4. South Asia | 2.6 (423) |
| 5. Vietnam | 0.4 (61) |
| *All* | *(26.6 (4 298)* |
|  |  |

**Table S2. Cox regression of use of psychiatric health care services among foreign-born refugee children by level of care**

|  | | |  |  | |  |
| --- | --- | --- | --- | --- | --- | --- |
|  | | |  | Model 1^a^  HR (95% CI) | Model 2^b^  HR (95% CI) | Model 3^c^  HR (95% CI) |
| MOTHER | | |  |  |  |  |
|  | No posttraumatic stress | | | 1 | 1 | 1 |
|  | Red cross centre | | | 2.00 (1.33-2.99) | 2.14 (1.43-3.22) | 2.19 (1.46-3.28) |
|  | Specialist psychiatric care | | | 2.30 (1.86-2.85) | 2.32 (1.87-2.88) | 2.17 (1.75-2.69) |
|  | Primary care | | | 1.64 (1.20-2.23) | 1.62 (1.19-2.21) | 1.52 (1.12-2.08) |
|  | FATHER | | |  |  |  |
|  | No posttraumatic stress | | | 1 | 1 | 1 |
|  | Red cross centre | | | 0.93 (0.57-1.50) | 0.94 (0.59-1.53) | 0.94 (0.58-1.52) |
|  | Specialist psychiatric care | | | 1.23 (0.93-1.62) | 1.18 (0.89-1.56) | 1.20 (0.91-1.59) |
|  | Primary care | | | 0.86 (0.60-1.24) | 0.84 (0.58-1.21) | 0.84 (0.58-1.22) |
|  |  | | |  |  |  |
|  | |  | |  |  |  |

^a^ Model 1 adjusted for birth year and gender.
^b^ Model 2 is adjusted Model 2 adjusted additionally for parental educational level, duration of residence, and parental country of origin.

^c^ Model 3 adjusted additionally for single parent household.

**Table S3. Cox regression of use of psychiatric health care services in children with two birth parents recorded as residents in Sweden** (N=13 255)

|  | Model 1^a^ | Model 2^b^ | Model 3^c^ |
| --- | --- | --- | --- |
|  |  |  |  |
|  | HR (95% CI) | HR (95% CI) | HR (95% CI) |
| **Parental post-traumatic stress** |  |  |  |
| No post-traumatic stress | 1 | 1 | 1 |
| Father post-traumatic stress | 1.04 (0.81-1.33) | 1.06 (0.82-1.36) | 1.01 (0.79-1.30) |
| Mother post-traumatic stress | 2.18 (1.75-2.70) | 2.20 (1.77-2.74) | 2.04 (1.63-2.53) |
| Both parents | 1.44 (0.96-2.15) | 1.55 (1.03-2.32) | 1.52 (1.02-2.27) |
|  |  |  |  |

^a^ Model 1 adjusted for birth year and gender.
^b^ Model 2 adjusted additionally for parental educational level, duration of residence, and parental country of origin.
^c^ Model 3 adjusted additionally for single parent household.
